# Increased Chromosomal and Oxidative DNA Damage in Patients with Multinodular Goiter and Their Association with Cancer

**DOI:** 10.1155/2017/2907281

**Published:** 2017-03-08

**Authors:** Hamiyet Donmez-Altuntas, Fahri Bayram, Nazmiye Bitgen, Sibel Ata, Zuhal Hamurcu, Gulden Baskol

**Affiliations:** ^1^Department of Medical Biology, Faculty of Medicine, Erciyes University, Kayseri, Turkey; ^2^Department of Endocrinology and Metabolism, Faculty of Medicine, Erciyes University, Kayseri, Turkey; ^3^Department of Chemical Technology, Technical Sciences Vocational School, Aksaray University, Aksaray, Turkey; ^4^Department of Biochemistry, Faculty of Medicine, Erciyes University, Kayseri, Turkey

## Abstract

Thyroid nodules are a common clinical problem worldwide. Although thyroid cancer accounts for a small percentage of thyroid nodules, the majority are benign. 8-Hydroxy-2′-deoxyguanosine (8-OHdG) levels are a marker of oxidative stress and play a key role in the initiation and development of a range of diseases and cancer types. This study evaluates cytokinesis-block micronucleus cytome (CBMN-cyt) assay parameters and plasma 8-OHdG levels and their association with thyroid nodule size and thyroid hormones in patients with multinodular goiter. The study included 32 patients with multinodular goiter and 18 age- and sex-matched healthy controls. CBMN-cyt assay parameters in peripheral blood lymphocytes of patients with multinodular goiter and controls were evaluated, and plasma 8-OHdG levels were measured. The micronucleus (MN) frequency (chromosomal DNA damage), apoptotic and necrotic cells (cytotoxicity), and plasma 8-OHdG levels (oxidative DNA damage) were significantly higher among patients with multinodular goiter. Our study is the first report of increased chromosomal and oxidative DNA damage in patients with multinodular goiter, which may predict an increased risk of thyroid cancer in these patients. MN frequency and plasma 8-OHdG levels may be markers of the carcinogenic potential of multinodular goiters and could be used for early detection of different cancer types, including thyroid cancer.

## 1. Introduction

Thyroid nodules are a common clinical problem affecting numerous individuals worldwide. According to epidemiologic studies, the prevalence of palpable thyroid nodules is approximately 5% among women and 1% among men living in iodine-sufficient parts of the world [1–3]. High-resolution ultrasound can detect thyroid nodules in 19%–68% of randomly selected individuals, with higher frequencies in women and the elderly [4, 5]. Although the majority of thyroid nodules are benign, certain risk factors, such as a solid nodule, age over 70 years or below 20 years, a history of previous head and neck irradiation, male sex, and a history of familial thyroid cancer or multiple endocrine neoplasia syndrome, may increase the risk of developing cancerous thyroid nodules [1, 6–8].

It is now well established that malignancies are characterized by variable amounts of chromosomal damage and spontaneous formation of micronucleus (MN). The MN formation has also been used as a tool for cancer risk prediction, screening, diagnosis, and monitoring response to therapy [9–12]. The cytokinesis-block micronucleus cytome (CBMN-cyt) assay has been used as a comprehensive method to cytologically evaluate chromosomal instability or damage status, on the basis of the presence of MN, as a biomarker of chromosome breakage or loss; of nucleoplasmic bridges (NPBs), as a biomarker of misrepair of DNA strand breaks or telomere end fusions; and of nuclear buds (NBUDs), as a biomarker of elimination of amplified DNA or DNA repair complexes in cultured human and/or mammalian cells. This method also assessed the mitotic status (mononucleated, metaphase, anaphase, binucleated, and multinucleated cells) and the viability status (necrosis, apoptosis) of the cell [13, 14]. Therefore, this assay is an important tool for the measurement of chromosomal DNA damage (MN, NPBs, and NBUDs), cytotoxicity (necrotic and apoptotic cell ratios), and cytostasis (the proportion of mono-, bi-, and multinucleated cells; nuclear division index (NDI)) in human peripheral blood lymphocytes. Recently, the in vitro mammalian cell MN test protocol was endorsed by the Organisation for Economic Co-operation and Development (OECD) as a standard method for testing of chemicals (OECD 487 guideline) [15].

Oxidative stress plays an important role in the development of different diseases and affects the induction of DNA damage. 8-Hydroxy-2′-deoxyguanosine (8-OHdG), a product of DNA base modification produced by the oxidation of deoxyguanosine, is widely accepted as a marker of oxidative DNA damage and oxidative stress [16, 17]. Elevated levels of 8-OHdG in the urine, plasma, and serum samples of patients with a range of cancer types have previously been reported [18–22]. Similarly, urinary 8-OHdG levels were higher in patients with hypoactive thyroid nodules (single and multiple) diagnosed with Graves' disease, toxic multinodular goiter, and Hashimoto's thyroiditis than those in the controls [23].

There are limited studies on the association between oxidative DNA damage and thyroid nodules; however, there are no data available in the literature on plasma 8-OHdG levels and CBMN-cyt assay parameters in the lymphocytes of patients with multinodular goiter. The present study was conducted to evaluate chromosomal DNA damage, cytotoxicity and cytostasis status, and plasma 8-OHdG levels and their association with thyroid nodule size and thyroid hormones, in patients with multinodular goiter.

## 2. Materials and Methods

### 2.1. Patients and Controls

The study group consisted of 32 patients (30 women and 2 men) with multinodular goiter, with a mean age of 48.16 ± 14.37 years (range, 19–87 years), admitted to the Department of Endocrinology and Metabolism at the Erciyes University's Medical Faculty between November 2013 and December 2014. An ultrasound examination of the thyroid was performed to detect thyroid nodules, and fine needle aspiration biopsy specimens were cytologically examined. All patients with nodular thyroids had bilateral multiple nodules and were euthyroid. The cervical lymph nodes were not enlarged. The mean size of the thyroid nodules was 16.24 ± 10.94 mm (range, 3–42 mm). The control group consisted of 18 healthy individuals (15 women and 3 men), with a mean age of 41.94 ± 11.47 years (range, 22–64 years) and matched for socioeconomic status. All participants completed a standardized questionnaire to obtain relevant details of current health status, history, and lifestyle and to collect information on past medical history and drug and smoking habits. No participants had been exposed to potentially confounding factors, such as exposure to other ionizing radiation (radiographic examination or scintigraphy) within the preceding three months. No participants were on medication or had concomitant diseases, such as hypertension, diabetes mellitus, heart disease, or cancer. We excluded patients and control subjects who reported alcohol drinking, cigarette smoking, tea or coffee consumption (more than three cups/day), and specific dietary habits (including vegetarian diets and eating hot peppers) and subjects who had a history of occupational or environmental exposure to known genotoxic chemicals. Blood samples were obtained from patients to measure free thyroxine (fT4) and thyroid-stimulating hormone (TSH). Heparinized antecubital blood samples (3-4 mL) were obtained from patients and control subjects for whole-blood cultures of human lymphocytes and determination of plasma 8-OHdG levels.

The local ethics committee approved the study protocol (number 2010/46), and all subjects gave written informed consent. The study was conducted in accordance with the Declaration of Helsinki and local laws, depending on which afforded greater protection to the patients.

### 2.2. Whole-Blood Cultures of Human Lymphocytes

After informed consent was obtained, a venous blood sample (3-4 mL) was taken from all patients with multinodular goiter and from control subjects, using heparinized tubes. Approximately 0.4 mL of heparinized whole-blood samples was cultured for 72 h at 37°C in 5 mL of peripheral blood karyotyping medium, supplemented with 1.5% phytohaemagglutinin-M (PHA-M) to stimulate T-lymphocytes (all sourced from Biological Industries, Kibbutz Beit Haemek, Israel). To determine intraindividual differences, duplicate cultures were made for each patient and control at the specified time [24].

### 2.3. CBMN-cyt Assay

Forty-four hours after the cultures were initiated, the cells were blocked from entering cytokinesis by the addition of cytochalasin-B to each culture tube (Sigma-Aldrich, St. Louis, MO; final concentration, 3 *μ*g/mL) [14, 24]. The cultures were stopped at 72 h after initiation, treated with hypotonic solution (0.1 mol/L KCl) for 4 minutes, and fixed using two changes of methanol acetic acid (3 : 1) [14, 24–26]. The fixed cells were spread onto glass slides and stained with 5% Giemsa (Merck) in Sorensen's buffer for 10 minutes. To determine the intraindividual differences, the different slides of the two parallel cultures for each patient and control subject were prepared and evaluated. All slides were scored blindly by a Zeiss light optical microscope. A score was obtained for slides from each duplicate culture from two different scorers by identical microscopes. One thousand binucleated (BN) cells with two macronuclei surrounded by cytoplasm were scored from each patient and control subject. Every subject was assessed to determine the total number of MN, NPBs, and NBUDs per 1000 BN cells to determine chromosomal DNA damage effects. The frequencies of BN cells containing one or more MN, NPBs, and NBUDs were determined. The number of necrotic and apoptotic cells was scored in 1000 mononucleated cells to determine cytotoxicity [14, 24].

The number of mono-, bi-, tri-, and tetranucleated cells per 1000 viable mononucleated cells was scored in peripheral blood lymphocytes of all individuals to determine cytostatic effects. NDI was calculated according to the following formula: NDI = (*M*1 + 2*M*2 + 3*M*3 + 4*M*4)/*N*, where *M*1–*M*4 represent the number of cells with 1–4 nuclei and *N* is the total number of viable cells scored (excluding necrotic and apoptotic cells) [14, 24, 27].

### 2.4. Determination of 8-OHdG Levels

Two milliliters of heparinized blood samples for analysis of 8-OHdG was immediately centrifuged at 3000 rpm for 15 min at room temperature. The plasma was then stored in microtubes at −80°C until it was analyzed. The 8-OHdG levels in plasma were measured using an ELISA kit (Catalogue: NWK-8-OHdG02, Northwest Life Science Specialties, LLC, WA, USA), and an intra-assay coefficient of variation of the 8-OHdG assay was calculated at 5.9%. Plasma 8-OHdG levels were expressed in ng/mL. Calibration, curve fitting, and data analysis were performed according to the manufacturer's instructions.

### 2.5. Statistical Analysis

The data were analyzed using SPSS for Windows statistical package, version 15.0. Differences were considered statistically significant when *p* values were less than 0.05. Statistical comparisons of CBMN-cyt assay parameters and plasma 8-OHdG levels between patients with multinodular goiter and control subjects were performed using a nonparametric Mann–Whitney *U* test for two independent samples. Spearman's rho correlation analysis was used to assess the association between age, fT4, TSH, thyroid nodule size, plasma 8-OHdG levels, and CBMN-cyt assay parameters.

## 3. Results

The general characteristics of patients with multinodular goiter and healthy control subjects are set out in Table 1. CBMN-cyt assay parameters and plasma 8-OHdG levels in patients with multinodular goiter and healthy control subjects are shown in Table 2.

According to the CBMN-cyt assay parameters, patients with multinodular goiter had a higher level of MN frequency (chromosomal DNA damage), apoptotic and necrotic cells (cytotoxicity), and plasma 8-OHdG levels (oxidative DNA damage) than controls (*p* < 0.01, *p* < 0.01, *p* < 0.01, and *p* < 0.05, resp.) (Table 2). However, no statistically significant differences were detected in NPB and NBUD frequencies (other chromosomal DNA damage parameters) and frequency of BN cells and NDI values (cytostasis) between the patients with multinodular goiter and control subjects (*p* > 0.05, Table 2).

Patients with multinodular goiter were subdivided into the following groups by nodule size: 1 (<10 mm and ≥10 mm), 2 (<20 mm and ≥20 mm), and 3 (<10 mm, 10–19 mm, and ≥20 mm). However, no statistical differences were detected in CBMN-cyt assay parameters and plasma 8-OHdG levels among nodule sizes for each group (*p* > 0.05, Kruskal-Wallis test and Mann–Whitney *U* test) (data not shown).

**Figure 1:**
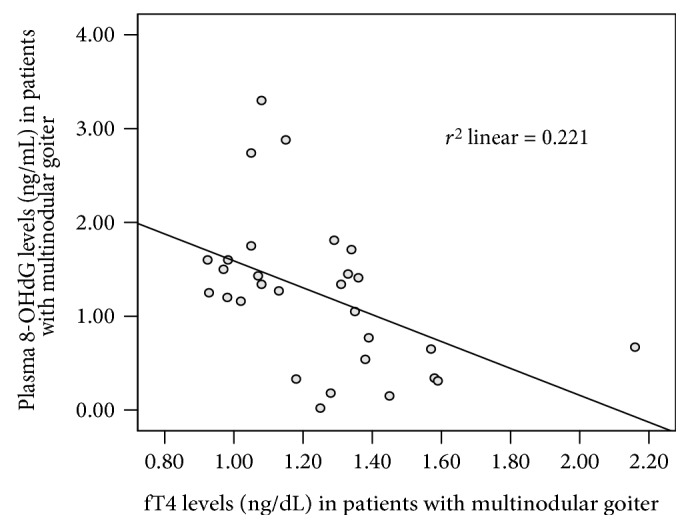
A negative correlation between fT4 and plasma 8-OHdG levels in patients with multinodular goiter (*p* < 0.01).

Table 3 shows Spearman's rho correlation coefficients and significance according to age, fT4, TSH, thyroid nodule size, plasma 8-OHdG levels, and CBMN-cyt assay parameters for patients with multinodular goiter and control subjects (Table 3). A negative correlation was observed between the following variables: thyroid nodule size and TSH (*p* < 0.05, *r*: −0.443; data not shown); fT4 and plasma 8-OHdG levels (*p* < 0.01, *r*: −0.512; Figure 1); age and NDI (*p* < 0.05, *r*: −0.411); and fT4 and TSH (*p* < 0.01, *r*: −0.472; data not shown). A positive correlation was found between thyroid nodule size and fT4 (*p* < 0.05, *r*: 0.482; data not shown) and between apoptotic cells and TSH (*p* < 0.05, *r*: 0.381), in patients with multinodular goiter (Table 3). In control subjects, negative correlation was observed between fT4 and plasma 8-OHdG levels (*p* < 0.05, *r*: −0.473) and a positive correlation was found between age and NPBs (*p* < 0.01, *r*: 0.640) and NBUDs (*p* < 0.05, *r*: 0.578) (Table 3).

## 4. Discussion

Thyroid nodules are common, with reported prevalence rates of 68% among the general population [1, 28]. Most thyroid nodules are benign, although in 7–15% of cases they are malignant, depending on age, sex, history of radiation exposure, family history, and other factors [1]. Cancer is a genomic disease, associated with the accumulation of genetic damage, and the majority of solid tumors show a large number of complex chromosomal aberrations. MN and other nuclear anomalies, such as NPBs and NBUDs, are biomarkers of genotoxic events and manifestations of chromosomal instability that are often found in cancer [9, 11]. The CBMN-cyt assay is one of the most widely used methods for the measurement of both structural and numerical chromosome abnormalities in lymphocytes. In addition, oxidative DNA damage is closely linked to carcinogenesis and 8-OHdG levels are a biomarker of oxidative DNA damage [16, 17, 29]. Hydrogen peroxide (H_2_O_2_) production was found in vivo in many intracellular structures, including the mitochondria, endoplasmic reticulum, and peroxisomes. H_2_O_2_ acts as an oxidant and also induces oxidative stress and apoptosis. It is known that thyroid hormone synthesis is associated with increased H_2_O_2_ production and free radical formation, which may damage genomic DNA and cause mutations [30, 31]. Therefore, oxidative stress and oxidative base adducts could contribute to the development of thyroid cancer [30, 32, 33]. However, the chromosomal and oxidative DNA damage, cytotoxicity, and the cytostasis status of patients with multinodular goiter remain unclear. The increased 8-OHdG levels may be the cause of the nodules occurring in patients with multinodular goiter. In this study, we have reported, for the first time, that chromosomal and oxidative DNA damage and frequency of apoptotic and necrotic cells were significantly higher in the lymphocytes of patients with multinodular goiter than in those of healthy controls. In addition, we showed a negative correlation between fT4 and plasma 8-OHdG levels, among patients and control subjects. One study [23] reported that urine 8-OHdG levels were significantly higher in the patients diagnosed with toxic multinodular goiter, Graves' disease, and Hashimoto's thyroiditis. Lymphocytes with genome damage in patients with multinodular goiter would either be eliminated by apoptosis or survive to express micronuclei in binucleated cells. Therefore, higher MN frequency and 8-OHdG levels in patients with multinodular goiters than in controls may result in an increase in apoptotic and necrotic cells (cytotoxicity).

The early molecular conditions for nodular and tumor transformation in the thyroid gland consist of a sequence of molecular events, including oxidative stress and DNA damage as the trigger for somatic mutations [31, 34]. Additionally, environmental conditions, such as iodine deficiency, may potentially aggravate this situation. An increased oxidative burden in the thyroid gland, through iodine deficiency, is suggested by results of the comet assay with repair-enzyme protocols to detect oxidative DNA damage in rats [35]. In general, any external factor, such as smoking, that increases oxidative stress, causes DNA damage, or increases spontaneous mutations, potentially increases the risk of tumorigenesis [31]. The patients and control subjects in our study were selected from nonsmoker volunteers, with no history of occupational or environmental exposure to known genotoxic chemicals, which may affect CBMN-cyt assay parameters and plasma 8-OHdG levels. Similarly, secondary factors, such as elevated TSH, may be involved in the development of multinodular goiters [30, 31]. However, we found that there was a poor association between apoptotic cells from CBMN-cyt assay parameters and TSH in patients with multinodular goiter. However, it is likely that all factors increasing proliferation shorten the time to develop a detectable thyroid tumor in patients with multinodular goiter [7, 31]. For example, estrogen is a potent growth factor for benign and malignant thyroid cells that may explain the sex difference in the prevalence of thyroid nodules and thyroid cancer. Estrogen and its receptors may play a pivotal role in the pathogenesis and progression of thyroid cancer in women [36].

Large nodule size has long been proposed as a risk factor for malignancy of thyroid nodules. Several studies indicate that nodules over 4 cm in size [37] or nodules over 2 cm in size [38] are more likely to be malignant than smaller nodules. However, in this study we did not observe significant differences in the CBMN-cyt assay parameters and plasma 8-OHdG levels among nodule sizes in the three groups of patients with multinodular goiter. Similarly, we found that the increases in plasma 8-OHdG levels and CBMN-cyt assay parameters (MN frequency, apoptotic, and necrotic cells) were not related to thyroid nodule size in patients with multinodular goiter. A negative correlation was observed between thyroid nodule size and TSH (*p* < 0.05, *r*: −0.443; data not shown), and a positive correlation was found between thyroid nodule size and fT4 (*p* < 0.05, *r*: 0.482; data not shown) in patients with multinodular goiter. This could be explained by the limited number of patients in our study.

There is conflicting evidence on the association between the size of nodules and the risk of malignancy [28, 37–40]. However, it is clear that thyroid nodule size on ultrasound is a poor predictor of malignancy when used alone for the assessment and management of thyroid nodules [37].

There are limitations to this study. The number of patients with multinodular goiter is small, and the lack of a large sample size may be an important bias when examining the association between thyroid nodule size and the parameters evaluated in this study. In addition, data on body mass index (BMI), a further risk factor for developing thyroid nodules, was not available.

To our knowledge, this is the first report of increased MN frequency, proportions of necrotic and apoptotic cells, and plasma 8-OHdG levels in patients with multinodular goiter. Data indicate that MN frequency in the general population predicts the increased risk of developing cancer, suggesting that increased MN frequency is associated with the early stages of carcinogenesis [9, 11]. Moreover, most studies suggest that increased levels of 8-OHdG and 8-oxoG reflect the early changes in the process of carcinogenesis, as a marker of oxidative DNA damage [29, 32].

It is important to note the high MN frequency and plasma 8-OHdG levels in patients with multinodular goiter, indicating increased chromosomal DNA and oxidative DNA damage or genome instability. These findings are potentially significant, as it is currently not known whether the benign nodular thyroid progresses, resulting in malignancy. Furthermore, accumulation of DNA mutations (higher replication rate or failure of mutation repair) may contribute to the pathogenesis of thyroid cancer [9, 11, 30–32]. Therefore, our results may indicate that monitoring of MN frequency and 8-OHdG levels is part of the necessary follow-up of patients with multinodular goiter in order to monitor the increased malignancy risk.

## 5. Conclusion

While the majority of thyroid nodules are benign, elevated chromosomal (MN frequency) and oxidative (8-OHdG levels) DNA damage are predictors of an increased risk of thyroid cancer and/or other types of cancer, in patients with multinodular goiter. Furthermore, MN frequency and plasma 8-OHdG levels may be markers of the carcinogenic potential of multinodular goiter and could be used for early detection of different cancer types, including thyroid cancer.

## Figures and Tables

**Table 1:** Characteristics of patients with multinodular goiter and control subjects (mean ± SD).

	Patients with multinodular goiter (*n* = 32)	Control subjects (*n* = 18)
Females (*n*)	30	15
Males (*n*)	2	3
Age (year)	48.16 ± 14.37	41.94 ± 11.47
Thyroid nodule size (mm)	16.24 ± 10.94	—
fT4 (ng/dL)	1.25 ± 0.26	1.31 ± 0.23
TSH (mIU/L)	2.04 ± 2.39	2.02 ± 0.74

fT4: free thyroxine; TSH: thyroid-stimulating hormone; and SD: standard deviation.

**Table 2:** CBMN-cyt assay parameters and plasma 8-OHdG levels in patients with multinodular goiter and control subjects (mean ± SD).

Parameter	Patients with multinodular goiter	Control subjects	*p* value (Mann–Whitney *U* test)
MN frequency (%)	1.90 ± 0.70	0.84 ± 0.50	0.001
NPB frequency (%)	0.55 ± 0.50	0.63 ± 0.29	0.108
NBUD frequency (%)	0.17 ± 0.11	0.20 ± 0.08	0.075
Frequency of apoptotic cells (%)	7.52 ± 6.38	1.17 ± 0.74	0.001
Frequency of necrotic cells (%)	7.20 ± 5.93	2.47 ± 0.79	0.001
Frequency of BN cells (%)	31.98 ± 15.44	27.90 ± 7.45	0.322
Nuclear division index (NDI)	1.27 ± 0.11	1.26 ± 0.08	0.746
Plasma 8-OHdG levels (ng/mL)	1.23 ± 0.81	0.67 ± 0.14	0.010

BN cells: binucleated cells; MN: micronucleus; NPBs: nucleoplasmic bridges; NBUDs: nuclear buds; 8-OHdG: 8-hydroxy-2′-deoxyguanosine; and SD: standard deviation.

NDI = [*M*1 + 2(*M*2) + 3(*M*3) + 4(*M*4)]/*N*, where *M*1–*M*4 represent the total number of lymphocytes with one to four nuclei scored on 1000 viable cells (excluding necrotic and apoptotic cells; *M*: the number of nuclei; *N*: the total number of viable cells scored).

**Table 3:** Spearman's rho correlation coefficient and significance values for age, TSH, fT4, and thyroid nodule size with CBMN-cyt assay parameters and plasma 8-OHdG levels in patients with multinodular goiter and control subjects.

	MN frequency (%)	NPB frequency (%)	NBUD frequency (%)	Frequency of apoptotic cells (%)	Frequency of necrotic cells (%)	NDI	Plasma 8-OHdG levels
Patients with multinodular goiter							
Age (years)							
*r*	−0.017	−0.133	0.065	0.205	0.214	−0.411^∗^	0.240
*p*	0.929	0.475	0.730	0.269	0.247	0.022	0.194
TSH (mIU/L)							
*r*	−0.089	0.029	−0.248	0.381^∗^	0.194	−0.049	0.318
*p*	0.646	0.880	0.195	0.042	0.312	0.802	0.093
fT4 (ng/dL)							
*r*	0.166	0.005	0.061	−0.197	−0.099	0.362	−0.512^∗∗^
*p*	0.388	0.980	0.755	0.306	0.611	0.053	0.005
Thyroid nodule size (mm)							
*r*	−0.243	0.105	0.110	−0.178	−0.114	−0.114	−0.279
*p*	0.276	0.642	0.628	0.428	0.615	0.615	0.209

Control subjects							
Age (years)							
*r*	0.176	0.640^∗∗^	0.578^∗^	0.053	−0.190	−0.339	0.319
*p*	0.485	0.004	0.012	0.833	0.449	0.169	0.197
TSH (mIU/L)							
*r*	0.142	−0.150	−0.354	0.075	0.424	−0.039	0.317
*p*	0.574	0.552	0.149	0.769	0.080	0.877	0.200
fT4 (ng/dL)							
*r*	0.342	−0.282	−0.322	−0.016	0.247	−0.153	−0.473^∗^
*p*	0.165	0.257	0.193	0.950	0.322	0.545	0.047

^∗^Correlation is significant at the 0.05 level, and ^∗∗^correlation is significant at the 0.01 level.

BN cells, binucleated cells; MN, micronucleus; NBUDs, nuclear buds; NPBs, nucleoplasmic bridges; NDI, nuclear division index; and 8-OHdG, 8-hydroxy-2′-deoxyguanosine.
